# Caffeine influence on the performance of the 5000m race

**DOI:** 10.1186/1550-2783-8-S1-P12

**Published:** 2011-11-07

**Authors:** Antonio FC Marangon, Paulo Henrique de Moraes Mendes

**Affiliations:** 1 Centro Universitário UniCEUB, Brazil

## Background

Caffeine is an ergogenic aid used to enhance athletic performance. It has central, intracellular and peripheral effects, which interfere on the prolongation and perception of fatigue during exhaustive efforts. Moreover, it hones and optimizes the cardiovascular, the endocrine, the muscular and the central nervous systems. Thus, its utilization can reduce the time of racing for tri-athletes and other sportsperson.

## Methods

With the purpose of investigating if caffeine influences the performance of tri-athletes during a 5000m race, nine male tri-athletes, aged between 18 and 35 years, participated in two tests of 5000 m time trials separated by an average of seven days. On one day they had a capsule containing caffeine anhydrous (5mg/kg), and on the other day they had a placebo capsule. The race timing on each time trial was monitored and blood samples were collected so as to measure blood glucose and blood lactate levels before and immediately after the end of each trial. A randomized double-blind study was used and analysis were carried out by using the t-student method, being determined significant values to p<0.05.

## Results

The average of the blood lactate before and after the trial on the caffeine group was 1.97 ±0.40 mmol/L and 4.46±1.16 mmol/L, respectively. On the placebo group, the average of the blood lactate before and after the trial was 2.21±0.31 mmol/L and 4.43±1.36 mmol/L, respectively. The average of the blood glucose level before and after the trial on the caffeine group was 108.33±15.1 mg/dL and 127±21.21 mg/dL, respectively. On the placebo group, the average of the blood glucose level pre and post trial was 107±12.5 mg/dL and 125±15.4 mg/dL, respectively. Thus, no statistically significant difference (p>0.05) was found on the results obtained from the blood glucose level and blood lactate between the caffeine and the placebo ingestion. However, a significant difference (p<0.05) in the mean time to complete each trial (caffeine vs. placebo trial) was observed with the caffeine trial in comparison to the placebo trial. It was obtained an average time of 21.39±3.1 min from the athletes using the placebo substance. As opposed to caffeine, the time was reduced to 20.48±3.15 min, showing a mean difference of 51±3.2 seconds between the caffeine and placebo trials.

## Conclusions

From the results analysis, it is possible to affirm that caffeine can be a powerful ergogenic resource, and that it can show beneficial effects on the aerobic performance, associated mainly to continuous long term activities. However, more studies are necessary in order to define and quantify precisely the factors that originate such influence on the performance.

